# Diabetes related phenotypes and their influence on outcomes of patients with corona virus disease 2019 (COVID-19)

**DOI:** 10.1186/s13098-023-01168-w

**Published:** 2023-10-16

**Authors:** Lais Isidoro Alves, Adriana Aparecida Bosco, Adriana Aparecida Rosa, Marcia Regina Soares Correia, Sergio Russo Matioli, Maria Elizabeth Rossi da Silva, HCFMUSP COVID-19 Study Group, Tarcisio E.P. Barros-Filho, Edivaldo M. Utiyama, Aluisio C. Segurado, Beatriz Perondi, Amanda C. Montal, Leila Harima, Solange R.G. Fusco, Marjorie F Silva, Marcelo C. Rocha, Izabel Cristina Rios, Fabiane Yumi Ogihara Kawano, Maria Amélia de Jesus, Esper Kallas, Maria Cristina Peres Braido Francisco, Carolina Mendes do Carmo, Clarice Tanaka, Maura Salaroli Oliveira, Thaís Guimarães, Carolina dos Santos Lázari, Marcello M.C. Magri, Julio F.M. Marchini, Alberto José da Silva Duarte, Ester C. Sabino, Silvia Figueiredo Costa, Anna Miethke Morais, Heraldo Possolo de Souza, Carlos Roberto Ribeiro Carvalho, Juliana Carvalho Ferreira, Anna Sara Shafferman Levin

**Affiliations:** 1 Laboratório de Carboidratos e Radioimunoensaio LIM-18, Hospital das Clínicas da Faculdade de Medicina da Universidade de São Paulo, São Paulo, Brazil; 2 Departamento de Genética e Biologia Evolutiva, Instituto de Biociências da Universidade de São Paulo, São Paulo, Brazil

**Keywords:** Diabetes, COVID-19, Clinical data, Laboratory data, Outcome

## Abstract

**Introduction:**

Diabetes mellitus (DM) is associated with severe forms of COVID-19 but little is known about the diabetes—related phenotype considering pre-admission, on-admission and data covering the entire hospitalization period.

**Methods:**

We analyzed COVID-19 inpatients (n = 3327) aged 61.2(48.2–71.4) years attended from March to September 2020 in a public hospital.

**Results:**

DM group (n = 1218) differed from Non-DM group (n = 2109) by higher age, body mass index (BMI), systolic blood pressure and lower O2 saturation on admission. Gender, ethnicity and COVID-19-related symptoms were similar. Glucose and several markers of inflammation, tissue injury and organ dysfunction were higher among patients with diabetes: troponin, lactate dehydrogenase, creatine phosphokinase (CPK), C-reactive protein (CRP), lactate, brain natriuretic peptide, urea, creatinine, sodium, potassium but lower albumin levels. Hospital (12 × 11 days) and intensive care unit permanence (10 × 9 days) were similar but DM group needed more vasoactive, anticoagulant and anti-platelet drugs, oxygen therapy, endotracheal intubation and dialysis. Lethality was higher in patients with diabetes (39.3% × 30.7%) and increased with glucose levels and age, in male sex and with BMI < 30 kg/m2 in both groups (obesity paradox). It was lower with previous treatment with ACEi/BRA in both groups. Ethnicity and education level did not result in different outcomes between groups. Higher frequency of comorbidities (hypertension, cardiovascular/renal disease, stroke), of inflammatory (higher leucocyte number, RCP, LDH, troponin) and renal markers (urea, creatinine, potassium levels and lower sodium, magnesium) differentiated lethality risk between patients with and without diabetes.

**Conclusions:**

Comorbidities, inflammatory markers and renal disfunction but not Covid-19-related symptoms, obesity, ethnicity and education level differentiated lethality risk between patients with and without diabetes.

**Supplementary Information:**

The online version contains supplementary material available at 10.1186/s13098-023-01168-w.

## Introduction

In the last two years, after the impact of a pandemic with immeasurable reflections on economic and social parameters and on public health, COVID-19 has become a routine care in the health systems. All the learning and controversies provided by the scientific communities allowed the knowledge of risk factors and tools necessary for better management and control of the infection. Demographic, ethno-social and gender characteristics were very similar in most countries, indicating that individuals of advanced age (especially over 65 years of age), male sex and belonging to ethnic and socio-economic minority groups were at greater risk for infectivity and worse outcomes. Comorbidities conditioning higher risk for severity of COVID-19 (hypertension, diabetes, cardiovascular disease and obesity), associated with insulin resistance, oxidative stress and vascular damage caused by low-grade inflammation that permeate all these comorbidities usually present worldwide were exacerbated by sedentary lifestyle, inadequate nutrition and the stress of modern life, worsened by the lockdown [1–8]. In addition, individuals with weaknesses and vulnerability such as chronic renal failure, cancer and those immunocompromised also evolved with several complications. All these factors favored a high rate of hospitalization and death, including the Brazil population [9–13].

SARs-CoV-2, the etiological agent responsible for severe acute respiratory syndrome (SARS), after binding to the angiotensin-converting enzyme-2 receptor (ACE-2), leads to endothelial dysfunction in the alveolar system and pulmonary endothelial cells, hindering the hematosis process, but also intensifies the systemic oxidative stress, insulin resistance and inflammation [14].

Patients with diabetes (DM) have been referred to as being at high risk for worse outcomes, expressed by exacerbated inflammatory response, tissue damage, thrombotic processes, greater need for mechanical ventilation and intensive care and higher mortality rate [15]. A more severe progression of COVID-19 described in patients who develop sustained hyperglycemia at the beginning of the infection, even in individuals without diabetes, were related to the systemic inflammation, insulin resistance and its vascular consequences, usually observed in individuals with diabetes or metabolic syndrome [6, 16].

Brazil is a socioeconomically and ethnically diverse country. The severe impact of COVID-19, was sensed in São Paulo, the first state to present infection by SARs-CoV-2 in Brazil and, due to its cosmopolitan characteristics, has become an important focus of the disease. Hospital de Clínicas da Faculdade de Medicina da Universidade de São Paulo (FMUSP), the largest academic hospital from Brazil become a reference for severely affected cases of COVID-19 in 2020.

Although the disproportionate impact of COVID-19 on patients with diabetes had already been evidenced, quantitative or qualitative studies assessing the clinical data and outcomes inequities in relation to those without diabetes were little evaluated or restricted to limited periods of time, [6, 7, 17]. This retrospective study provide details covering the entire period of hospitalization, focusing on log-term prognosis. We analyzed demographic characteristics, ethnicity, educational level, clinical and laboratory parameters on admission and during the final progression to fatal or non-fatal outcomes, as well as the impact of previous comorbidities in patients with diabetes in relation to the population without diabetes.

## Research design and methods

The study was approved by the Ethical Committee of Hospital das Clinicas, Faculdade de Medicina, Universidade de São Paulo (CAAE 53303621.0.0000.0068), and followed the guidelines of the Declaration of Helsinki. Informed consent was obtained from all patients.

Only cases of hospitalized patients affected by SARS-CoV-2, aged 18 years and over consecutively admitted to the hospital were considered in this descriptive, retrospective and cross-sectional study, carried out through a review of data collected of 3778 patients with severe acute respiratory syndrome attended in the period from March to September 2020. Of these, 426 patients who tested negative for SARs-COV-2 (RT-PCR or serological tests) or did not meet the epidemiological criteria for COVID and 25 patients youger than 18 years were excluded. We analyzed 3009 patients positive for COVID-19 plus 318 patients with very high clinical suspicion and/or radiological information who did not undergo the test, totaling 3327 patients.

## Statistical analysis

The distributions of demographic data were verified by Shapiro–Wilk normality test. Numerical variables with parametric and non-parametric distribution were analyzed by unpaired Student's t-test and Mann–Whitney test, respectively. The correlations were performed by Pearson's correlation coefficient or Spearman's rank correlation coefficient. Continuous and categorical variables were presented as median interquartile range (IQT) and n (%). Qualitative variables were compared using the Chi-square test or the Fisher’s exact test, with the statistical package Graph Pad Prism. To control multiplicity in confirmatory results we used a so-called hierarchical, or fixed sequence, test procedure to determine the significant p value [18].

Besides individual comparisons between variables of the diabetic and non-diabetic groups, we also performed a principal component analysis. In this analysis, the original variables were mathematically transformed in its principal components, i.e., new variables that contains all variation of the original variables but that no longer present correlations among them. The principal component variables that comprise at least 50% of all variation were further compared between the groups and were also used in a logistic regression to verify its predictive power on the outcome.

The principal component analyses were done in four groups, according to physiological criteria. The first group comprised standard measures on admission and the resulting principal components were named Adm PC 1 and Adm PC 2 for further analyses.

The second, third and fourth groups comprised laboratory analyses related to inflammatory, coagulation and renal status and the resulting principal components were named INFLA PC 1 and INFLA PC 2, COAG PC 1 and COAG PC 2 and RENAL PC 1 and RENAL PC 2 respectively.

For each group we compared their principal components between patients with and without diabetes, between dead and released patients (in the total or separated by diabetes status).

Principal components variables were compared by the Chi square approximation after Wilcoxon non-parametric test for principal components that have variances significantly different as verified by the Levene test. When the variances were not significantly different after the Levene test, the t test was applied. The predictive power of the principal components for death/release outcomes were estimated by logistic regression and receiver operating characteristic curves (ROC).

Missing data were reported in tables and supplementary material.

## Results

Data are presented as numbers (%) or median (25–75th percentile).

Among the 3327 individuals admitted to the hospital, prevailed those self-reported as white (64.98%), males (55.4%) and with basic educational level (68.4%). The median age was 61.2 years (48.2–71.4) and body mass index (BMI) was 26.30 kg/m^2^ (23.4–31.1). Upon admission, the most frequent comorbidities were arterial hypertension (57.4%) followed by diabetes mellitus (36.6%), cardiovascular disease (CVD- 20.6%), obesity (24.7%), cancer (13.9%), chronic kidney disease and/or dialysis (CKD) (11.67%). In smaller proportions, were chronic obstructive pulmonary disease (9.8%), stroke (7.13%), neurological (8.9%) and gastrointestinal diseases (4.42%).There was a predominance of ex-smokers (21.13% × 6.42%) (Additional file 1: Table S1).

Diabetes was the second most common comorbidity in the hospitalized population. These patients (DM group; n = 1218) differed significantly from those without diabetes (Non-DM group; n = 2109) in terms of higher age and BMI and lower educational level (Table 1). As expected, individuals with diabetes also presented a higher frequency of the following comorbidities: CVD, hypertension, previous stroke, CKD/dialysis and obesity. Many of them were ex-smokers and used ACE inhibitors and/or angiotensin II receptor blockers (ACEI/BRA) medications more frequently. On the other hand, cancer predominated among individuals without diabetes. There was no difference between groups regarding sex and ethnicity and in the frequency of previous pulmonary, gastro-intestinal and neurological diseases.

**Table 1:** Clinical data of COVID-19 patients on hospital admission

Data at admission	DM Group		Non-DM Group		DM Group		Non-DM Group		OR	CI	P value	Critical p value
Variables (N = 37)	Median	IQR	Median	IQR	(%)	Positive/total	(%)	Positive/total				
Hypertension, %					82.32	1001/1216	42.98	906/2108	6.177	5.207–7.327	** < 0.0001**	0.00135
Age (years)	65.15	56.1–73.4	57.9	43.7–69.7							** < 0.0001**	0.00139
RAAS inhibitor drug, %					54.46	513/942	34.14	492/1441	2.307	1.949–2.729	** < 0.0001**	0.00143
Cardiovascular disease, %					28.36	344/1213	16.09	339/2107	2.065	1.741–2.449	** < 0.0001**	0.00157
Stroke, %					10.67	130/1218	5.08	107/2108	2.234	1.712–2.916	** < 0.0001**	0.00152
SAPS 3	68	55–79	63	51–74							** < 0.0001**	0.00156
Renal failure or dialysis, %					15.44	188/1218	9.49	200/2108	1.74	1.407–2.155	** < 0.0001**	0.00161
Systolic blood pressure, mmHg	127	110–140	120	110–135							** < 0.0001**	0.00167
Body mass index, kg/m2	27.12	24.30–31.59	25.95	22.86–30.48							** < 0.0001**	0.00172
Cancer, %					10.52	121/1150	15.97	302/1891	0.6187	0.4940–0.7749	** < 0.0001**	0.00179
Obesity, %					33.99	414/1218	27.03	570/2109	1.390	1.193–1.629	** < 0.0001**	0.00185
Previous smoker, %					25.10	304/1211	18.85	396/2101	1.443	1.218–1.710	** < 0.0001**	0.00192
Low education level, %					74.96	404/539	64.26	543/845	1.652	1.332–2.048	** < 0.0001**	0.00200
Fever, %					52.72	620/1176	58.80	1186/2017	0.7813	0.6760 -0.9031	**0.0009**	0.00208
Oxigen saturation, %	94	91–96	94	91–97							**0.0015**	0.00217
Tiredness, %					32.90	355/1079	27.66	492/1779	1.283	1.089–1.511	0.0033	0.00227
Temperature ^0C^	36.1	36–37	36.15	36–37							0.0042	0.00238
Headache, %					17.07	197/1154	21.37	418/1956	0.7574	0.6278–0.9138	0.0042	0.00250
Muscle ache, %					28.60	332/1161	33.03	653/1977	0.8120	0.6932–0.9511	0.011	0.00263
Smell loss, %					12.45	144/1157	15.59	306/1963	0.7698	0.6221–0.9525	0.0182	0.00278
Respiratory rate, breaths/min	24	20–28	23	20–28							0.07229	0.00294
Heart rate, beats/min	88	77–99	88	78–100							0.0881	0.00313
Male sex, %					53.53	652/1218	56.42	1190/2109	0.8896	0.7720–1.025	0.1057	0.00333
Taste loss, %					13.49	156/1156	15.34	300/1956	0.8611	0.6988–1.061	0.1764	0.00357
Stroke or confusion, %					9.64	99/1027	8.28	141/1703	1.182	0.9025–1.548	0.25	0.00385
Cough, %					71.17	842/1183	69.58	1400/2012	1.079	0.9219–1.264	0.3428	0.00417
Gastrointestinal/ liver disease, %					4.84	59/1218	4.18	88/2107	1.168	0.8332–1.637	0.373	0.00455
White ethnicity, %					65.85	781/1186	64.43	1299/2016	10064	0.9154–1.238	0.417	0.00500
Symptoms duration (days)	9	6.0–12	9	6.0–12							0.4806	0.00556
Diastolic blood pressure, mmHg	75	65–84	75	67–83							0.4888	0.00625
Runny nose, %					11.81	122/1033	12.78	219/1714	0.9142	0.7218–1.158	0.4935	0.00714
Smoker current, %					6.00	73/1217	6.66	140/2103	0.8947	0.6680–1.498	0.5009	0.00833
Neurological disease/dementia, %					8.49	88/1037	9.18	142/1547	0.9175	0.6945–1.212	0.54	0.01000
Dyspnea, %					77.49	919/1186	76.68	1555/2028	1.047	0.8826–1.242	0.6288	0.01250
Gastrointestinal complaints, %					22.47	262/1166	23.10	456/1974	0.9648	0.8116–1.147	0.7171	0.01667
Chronic lung disease/asthma, %					10.02	122/1218	9.68	204/2108	1.039	0.8202–1.316	0.7978	0.02500
Odionophagy, %					7.11	82/1154	7.05	137/1942	1.008	0.7587–1.339	0.985	0.05000

DM, diabetes mellitus; SAPS 3, Simplified Acute Physiology III score; OR, odds rate; CI, confidence interval; Critical p value, p value adjusted by number of variables

Considering the characteristics prior to hospital admission, the median duration of COVID-19 symptoms (9 days), as well as the frequency of the following complaints were similar between groups: dyspnea (the most frequent symptom—near 77% in both groups), cough (70%), runny nose, odynophagia, headache, muscle pain, anosmia, ageusia, mental confusion, tiredness and gastrointestinal complains. Fever before hospitalization was less frequent in patients with diabetes than in those without diabetes (Table 1).

On hospital admission, patients with diabetes presented lower O2 saturation, higher systolic blood pressure and higher Score Simplified Acute Physiology III (SAPS3) index (suggestive of a worse prognosis for the evolution). The body temperature, heart and respiratory rate and the diastolic blood pressure were similar in both groups (Table 1).

As expected, glucose levels on admission were higher among patients with diabetes, as well as several markers of inflammation, tissue injury and organ dysfunction like troponin, lactate dehydrogenase (LDH), creatine phosphokinase (CPK), C-reactive protein (CRP), lactate and brain natriuretic peptide (BNP) levels. Likewise, markers of kidney damage were more prevalent among patients with diabetes, evidenced by greater blood levels of urea, creatinine, sodium, potassium and lower of albumin. Only aspartate aminotransferase (AST), alanine aminotransferase (ALT) and magnesium were lower in these patients (Table 2).

**Table 2:** Laboratory data of patients with COVID-19 on-admission

Laboratory Data	Diabetes		Non-Diabetes		P value	Critical P value
Variables (N = 23)	Group median	IQR	Gtoup	IQR		
			median			
Glucose mg/dL	214.00	143–302	127	104–165	** < 0.0001**	0.002174
ureia, mg/dL	56.00	34–93	40	26–69	** < 0.0001**	0.002273
potassium, mEq/L	4.30	3.9–4.8	4.2	3.8–4.7	** < 0.0001**	0.002381
magnesium,mg/dL	2.02	1.82–2.28	2.09	1.88–2.32	** < 0.0001**	0.0025
aspartate aminotransferase, U/L	36.00	25–58	41	27–65	** < 0.0001**	0.002632
alanine aminotransferase, U/L	30.00	20–48	34	21–58	** < 0.0001**	0.002778
creatinine, mg/dL	1.14	0.8–2.0075	0.94	0.7–1.4925	** < 0.0001**	0.002941
sodium, mEq/L	139.00	136–142	139	137–142	** < 0.0001**	0.003125
troponin, ng/mL	0.025	0.012–0.074	0.02	0.007–0.044	** < 0.0001**	0.003333
B-type natriuretic peptide, pg/mL	1061.00	303.5–4005	574	150–2973	** < 0.0001**	0.003571
creatine phosphokinase, U/L	140.00	58–460.5	107.5	51–304.75	**0.0002**	0.003846
lactate, mmol/L	14.00	11.0–18	13	10.0–18	**0.0004**	0.004167
C-reactive protein, mg/L	129.75	68.025–225.6	111.55	55.6–214.05	**0.0005**	0.004545
Albumin, g/dL	3.00	2.6–3.3	3.1	2.7–3.4	**0.0016**	0.005
lactate dehydrogenase, U/L	429.00	309–569.5	391	290–541	**0.0017**	0.005556
fibrinogen,mg/dL	551.00	434.75–664	525	396–664	0.0815	0.00625
hemoglobin g/dL	12.00	10.5–13.4	12.2	10.4–13.6	0.0936	0.007143
leucocytes, 109/L	8.81	6.31–12.44	8.55	5.98–12.27	0.1459	0.008333
D-dimer ng/mL	1548.50	822.75–4431.5	1476.5	785.5–4359.75	0.2269	0.01
Platelet, 10 ^9^/L	225.00	165–301	223.5	163–297	0.5662	0.0125
prothrombin time, seconds	12.80	12.0–14	12.9	12–14.1	0.781	0.016667
partial thromboplastin time, seconds	29.90	27.1–33.5	29.9	27.1–33.3	0.8246	0.025
ionic calcium, mg/dL	4.68	4.47–4.89	4.68	4.48–4.86	0.9238	0.05

IQR, interquartile range; Critical p value, p value adjusted by number of variables

No difference was observed between groups in hemoglobin levels, leukocyte count or clotting markers like D-dimer, prothrombin time (PT), partial thromboplastin time (PTP), fibrinogen levels, platelet number and ionic calcium.

Disease severity was great in both groups, considering the necessity of medications and intensive care, which were higher in patients with diabetes (69.9% × 60.4%) (Table 3. This was also evidenced by their greater need of vasoactive, anticoagulant and anti-platelet drugs, oxygen therapy, endotracheal intubation and dialysis. They also received ACEI/BRA more frequently (34.3% × 22.7%; p < 0.0001). No difference between groups was observed for the frequency of blood transfusion, and treatment with glucocorticoids, antibiotics, anti-viral, anti-fungal and immunosuppressive drugs.

**Table 3:** Treatment data during hospitalization of COVID-19 patients

Treatment Data	Diabetes group		Non-diabetes group		Diabetes group		Non-diabetes group		OR	CI	P value	Critical P value
Variables (N = 28)	median	IQR	median	IQR	(%)	Positive/total	(%)	Positive/total				
Antiplatelet, %					28.98	353/1218	13.33	281/2108	2.653	2.224–3.165	< 0.0001	0.00179
RAASi, %					34.32	418/1218	22.66	478/2109	1.783	1.525–2084	< 0.0001	0.00185
Vasoactive drug, %					60.25	676/1122	49.36	925/1874	1.555	1.338–1.807	< 0.0001	0.00192
Oxygen therapy, %					94.37	1139/1207	88.93	1847/2077	2.086	1.576–2.761	< 0.0001	0.00200
Intensive care unit, %					69.95	852/1218	60.36	1273/2109	1.529	1.315–1.777	< 0.0001	0.00208
Intubation, %					59.49	677/1138	50.45	960/1903	1.443	1.243–1.674	< 0.0001	0.00217
Dialysis, %					24.79	302/1218	19.25	406/2109	1.383	1.168–1.638	0.0002	0.00227
Anticoagulant drug, %					98.11	1195/1218	95.78	2020/2109	2.289	1.439–3.641	0.0005	0.00238
Hospital stay (days)	12.00	7–20	11.00	6–19							0.0274	0.00250
Anticoaguant drug duration (days)	10.97	5.79–19.24	10.13	5.31–18.39							0.0549	0.00263
Antiviral drug, %					32.35	394/1218	35.63	751/2108	0.8640	0.7440–1.003	0.0552	0.00278
Antifungal drug n = 269, %					9.28	113/1218	7.40	156/2108	1.280	0.9934–1.648	0.0648	0.00294
Dialysis duration (days)	6.00	2–13	7.00	2–16							0.0912	0.00313
Antibiotic drug duration (days)	5.42	2.63–13.27	5.20	2.39–12.53							0.1344	0.00333
RAASi drug duration	5.38	2.10–10.02	4.96	1.86–9.73							0.1819	0.00357
Antiviral drug duration (days)	1.71	0.50–3.62	1.84	0.50–3.68							0.2249	0.00385
Immunossuppressordrug, %					3.94	48/1218	3.23	68/2108	1	0.8446- 1.793	0.3247	0.00417
Antibiotic drug, %					92.36	1125/1218	91.46	1928/2108	1.129	0.8700–1.466	0.3959	0.00455
Immunosuppressor duration (days)	5.74	2.62–13.06	5.95	2.47–14.41							0.4272	0.00500
Antiplatelet drug duration (days)	7.42	3.45–13.78	6.29	2.20–12.89							0.4691	0.00556
Endotraqueal entubation duration (days)	10.00	6.0–17	10.00	6.0–16							0.5349	0.00625
Glicocorticoid drug, %					62.07	756/1218	60.96	1285/2108	1.048	0.9065–1.212	0.5506	0.00714
Intensive Care Unit stay (days)	10.00	5–17	9.00	5–17							0.5872	0.00833
Vasoactive drug duration (days)	6.00	3–11	6.00	3–10							0.6657	0.01000
Antifungal drug duration (days)	3.76	1.00–6.62	5.40	1.32–9.72							0.9289	0.01250
Tracheostomy, %					12.59	85/675	12.43	119/957	1.015	0.7533–1.366	0.9395	0.01667
Glicocorticoid drug duration (days)	6.95	3.08–14.08	6.88	3.03–13.99							0.9917	0.02500
Blood transfusion, %					19.70	240/1218	19.63	414/2109	1.005	0.8413–1.200	0.9947	0.05000

IQR, interquartile range; OR, odds rate; CI, confidence interval; Critical p value, p value adjusted by number of variables RAASi, inhibitors of the renin- angiotensin-system sistem

**Fig. 1:**
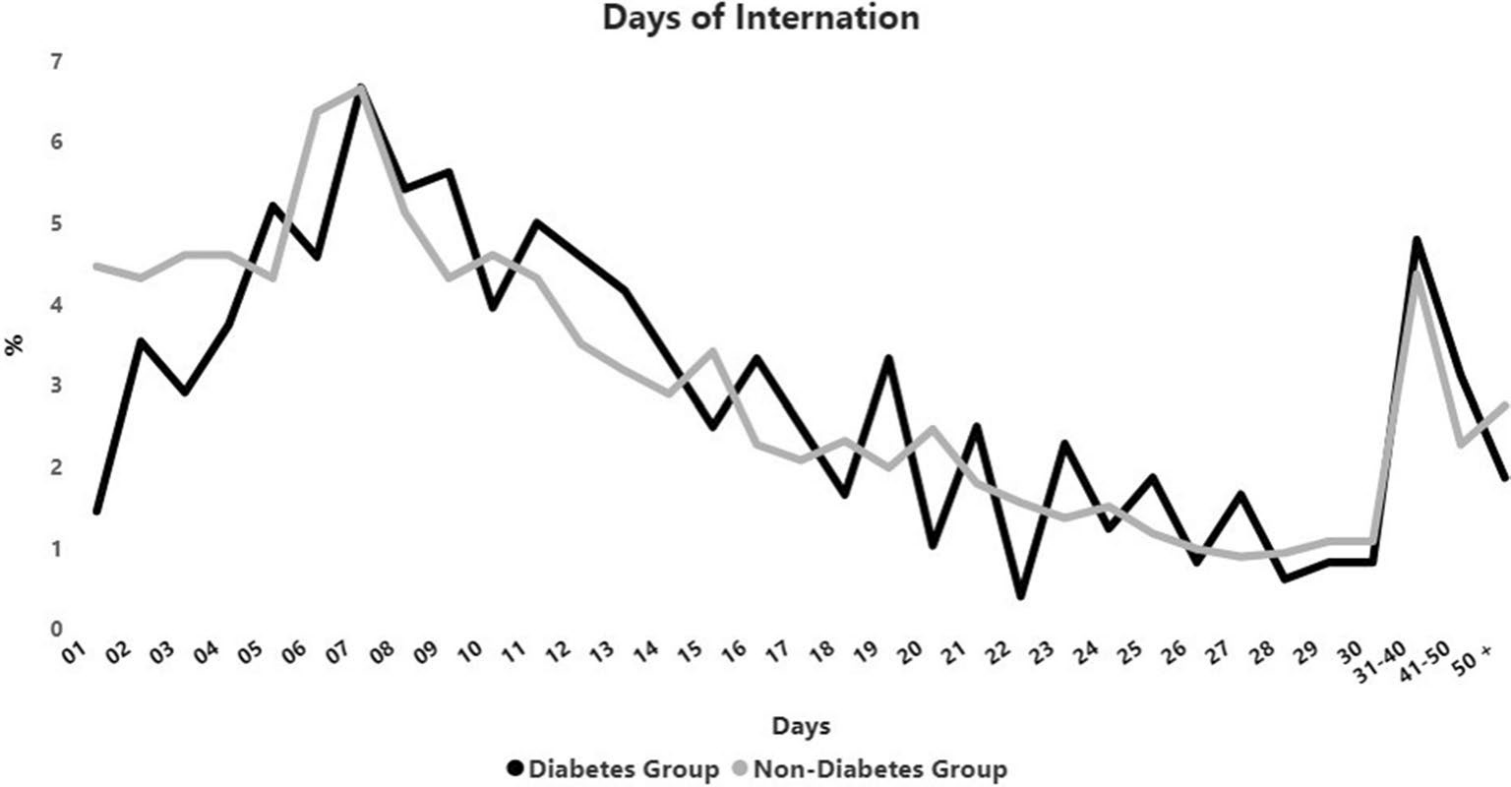
Duration of hospital permanence of patients with and without diabetes

On the other hand, the median duration of hospital (12 × 11 days) (Fig. 1) (Additional file 1: Table S2) and intensive care unit (ICU) permanence (10 × 9 days) as well as the duration of all reported therapies were similar between groups (Table 3).

Patients with diabetes had a higher fatality rate (39,3% × 30,7%) which predominated in men in both groups (Table 4).

**Table 4:** influence of sex and diabetes on fatality rate of COVID-19 patients

Group		Death frequency (%)	Positive/total	OR	IC	p	Critical P value
DM group		39.24	419/1218	1.46	1.260–1.693	< 0.00001	0.017
Non-DM group		30.72	648/2109				
Non-DM group	Male	34.5	410/1190	1.504	1.244–1.819	< 0.0001	0.025
	Female	25.9	238/919				
DM Group	Male	42.8	279/652	1.369	1.085–1.726	0.0082	0.05
	Female	35.3	200/566				

DM, diabetes mellitus; OR, odds rate; CI, confidence interval; Critical p value, p value adjusted by number of variables (n = 3)

The age (years) of death did not differ between women and men in both DM [67.60 (57.20–76.60) × 67.45 (59.50–75.23) p = 0.828] and non-DM group [64.7(52.3–75-38) × 66.95(56.48–74.83); p = 0.360].

In both groups the unfavorable outcome was observed in those presenting higher age and cardiac rate, lower systolic and diastolic blood pressure levels and lower BMI on admission. Higher blood levels of glucose, CRP, LDH, AST, ALT, D-dimer, PT, CPK, lactate, urea, creatinine, sodium, potassium, magnesium, troponin, BNP, Pt and leucocyte count and lower albumin levels and platelet count probably also contributed to the mortality in both groups. There were few differences between groups. Lower ionic calcium, higher lactate and PTT, and chronic renal disease were observed in patients with diabetes that evolved to death. Lower haemoglobin, fibrinogen levels and oxygen saturation and higher prevalence of male sex and hypertension were also associated with mortality in non-DM group (Additional file 1: Table S3–S8).

Several COVID-19 complaints like fever, cough, runny nose, odynophagia, headache, tiredness, muscle pain, anosmia, ageusia, and gastrointestinal complains on admission had lower frequency in those progressing to death. Dyspnea and mental confusion were similar (Additional file 1: Table S3, S6). All these complaints did not differ between patients with and without diabetes (Additional file 1: Table S9).

Comorbidities were frequent in both groups progressing to death, mainly in patients with diabetes, with almost twice the frequency of hypertension, CVD, CKD and previous stroke in relation to those without diabetes (Additional file 1: Table S9). They also presented higher age and higher glucose, troponin, PKC, urea, creatinine, sodium and potassium levels, needed more intensive care, anti-platelets and vasoactive drugs. Only cancer prevailed in those without diabetes (Additional file 1: Table S9–S11).

**Fig. 2:**
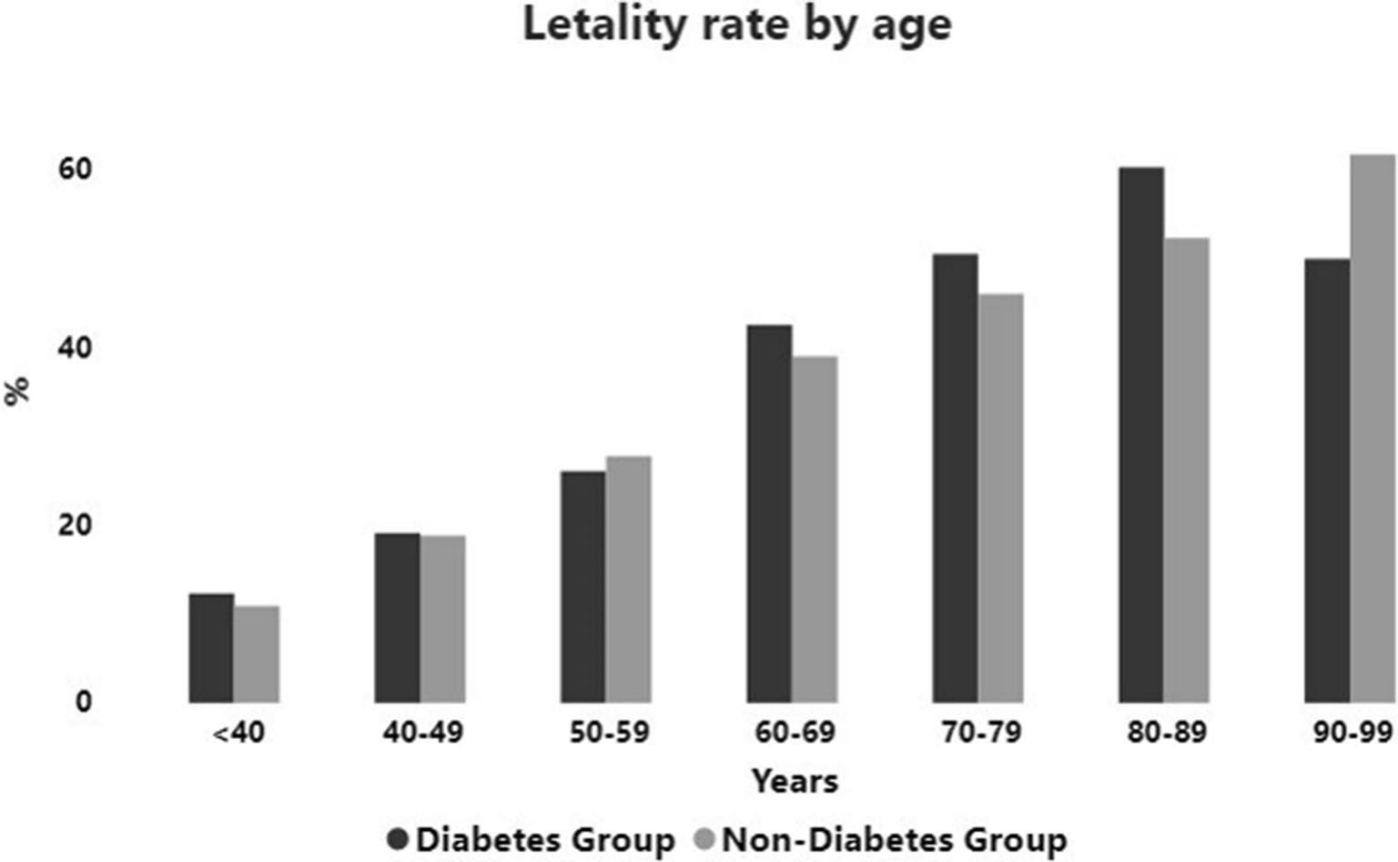
Lethality rate (%) by age at admission of patients with and without diabetes

In general, mortality increased with age. It was around 11–12% in younger than 40 to around 60% in patients over 80 and 90 years with and without diabetes respectively (Fig. 2). The frequency of death was similar between groups for all age ranges (Additional file 1: Table S12).

**Fig. 3:**
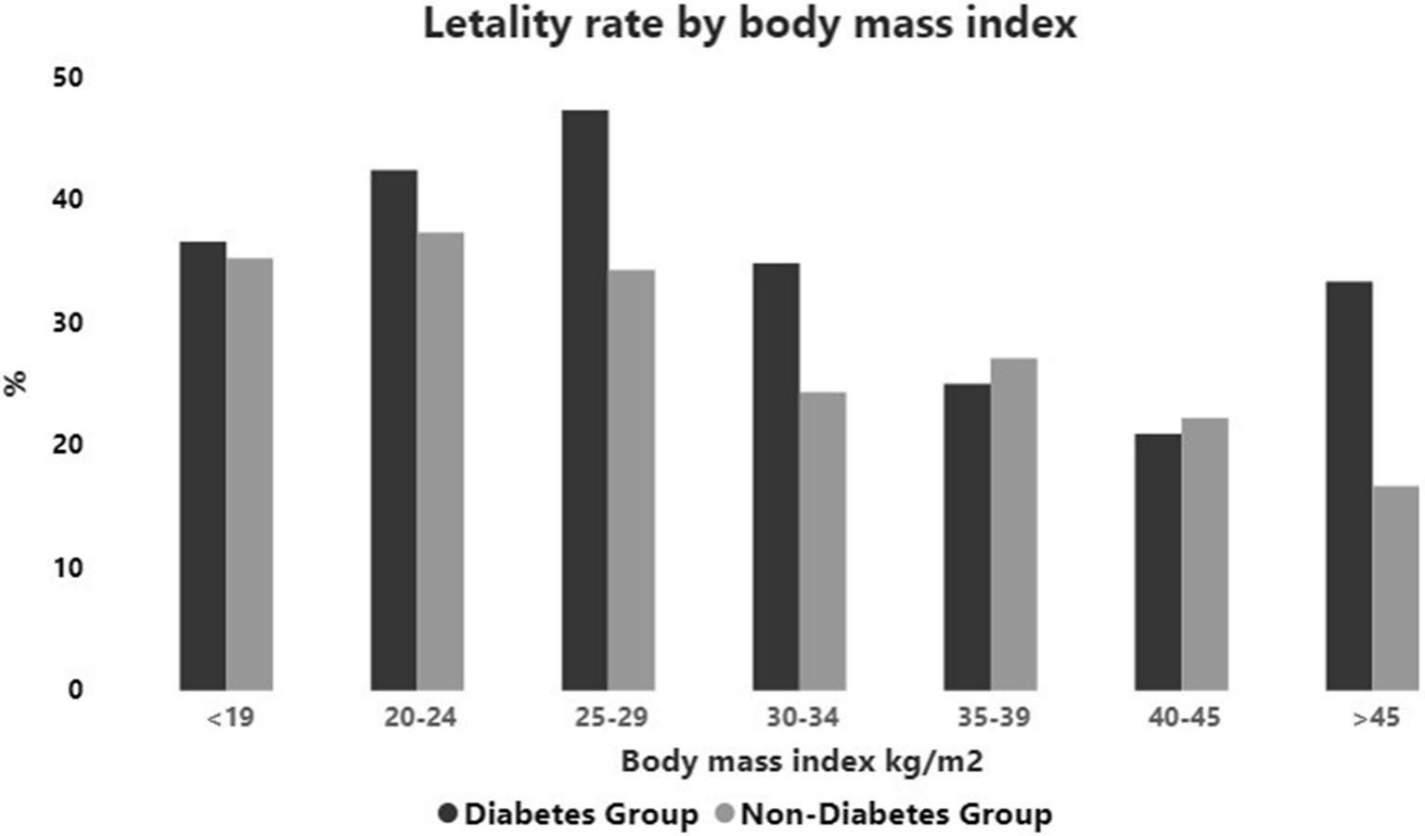
Lethality rate (%) by body mass index (kg/m2) at admission of patients with and without diabetes

The mortality rate prevailed in those with BMI lower than 30 kg/m2 in both groups (Additional file 1: Table S3, S6), being higher in patients with DM in relation to those without DM only for those with BMI between 25 and 29.9 kg/m2 (47.3 × 34.3%; OR = 1.72, CI 1.306–2.268; p < 0.0001) (Fig. 3) (Additional file 1: Table S13).

Ethnicity and education level did not result in different outcomes between groups. The frequency of death was similar between patients with and without diabetes for those with high and with low education level (Table 5).

**Table 5:** Influence of education level, ethnicity and treatment with renin–angiotensin–aldosterone blokers on lethalilty of patients with COVID-19

Variables N = 6	Characteristic	Group	Death %	Positive/total	OR	CI	P value	Crtical P value
Education level	Low	DM Group	57.67	233/404	1.189	0.9169–1.541	0.2094	0.025
		Non-DM Group	53.41	290/543				
	High	DM Group	53.33	72/135	1.512	1.006–2.273	0.0491	0.016667
		Non-DM Group	43.05	130/302				
Self reported skin colour	White	DM Group	39.26	307/ 782	1.490	1.237–1.795	** < 0.0001**	0.008333
		Non-DM Group	30.25	393/ 1299				
	Non-white	DM Group	40.99	166/405	1.443	1.121–1.857	**0.0052**	0.0125
		Non-DM Group	32.50	233/717				
ACEi/BRA treatment	Positive	DM Group	33.53	172/513	1.172	0.8986–1530	0.2501	0.05
		Non-DM Group	30.08	148/492				
	No	DM Group	48.95	210/429	1.597	1.268–2.011	** < 0.0001**	0.01
		Non-DM Group	37.51	356/949				

DM, diabete mellitus; ACEi/BRA, renin–angiotensin–aldosterone blokers; OR, odds rate; CI, confidence interval; Critical p value, p value adjusted by number of variables

No influence was also observed when comparing the frequency of deaths in those self-reported as white and non-white in DM group (39.26% × 40.99%; OR = 0.9305; CI 0.7286–1.188; p = 0.574) or non-DM group (30.2% × 32.5%; OR = 0.9011; CI 0.7406–1.096; p = 0.9011). The median age of death in whites was similar to of non-whites in both DM (69.5 × 69.6; p = 0.7613) and non-DM groups (65.9 × 66.3; p = 0.7924). Further, the lethality rate of patients with diabetes remained greater than those without diabetes for both those self-referred as white (39.26% × 30.25%; OR 1.490 (1.237–1.795); p < 0.0001) or non-white (40.99% × 32.50%; OR 1.443 (1.121–1.857); p = 0.0052) (Table 5).

Lethality was lower with previous treatment with ACEi/BRA in both DM group (33.5% × 48.95%; OR = 0.526; CI 0.4041–0.6846; p < 0.0001) and non-DM group (30.08% × 37.51%; OR = 0.7167; CI 0.5675–0.9049; p = 0.0052).

Further, previous use of ACEi/BRA seemed to be associated with protection to patients with diabetes. The lethality among DM and non-DM groups with this therapy was similar (33.53 × 30.08; OR 1.172; CI 0.8986–1.530; p = 0.2501) but, in those without ACEi/BRA, it was higher among patients with than those without diabetes (48,95% × 37.51%; OR 1.597; CI 1.268–2.011; p < 0.0001) (Table 5).

**Fig. 4:**
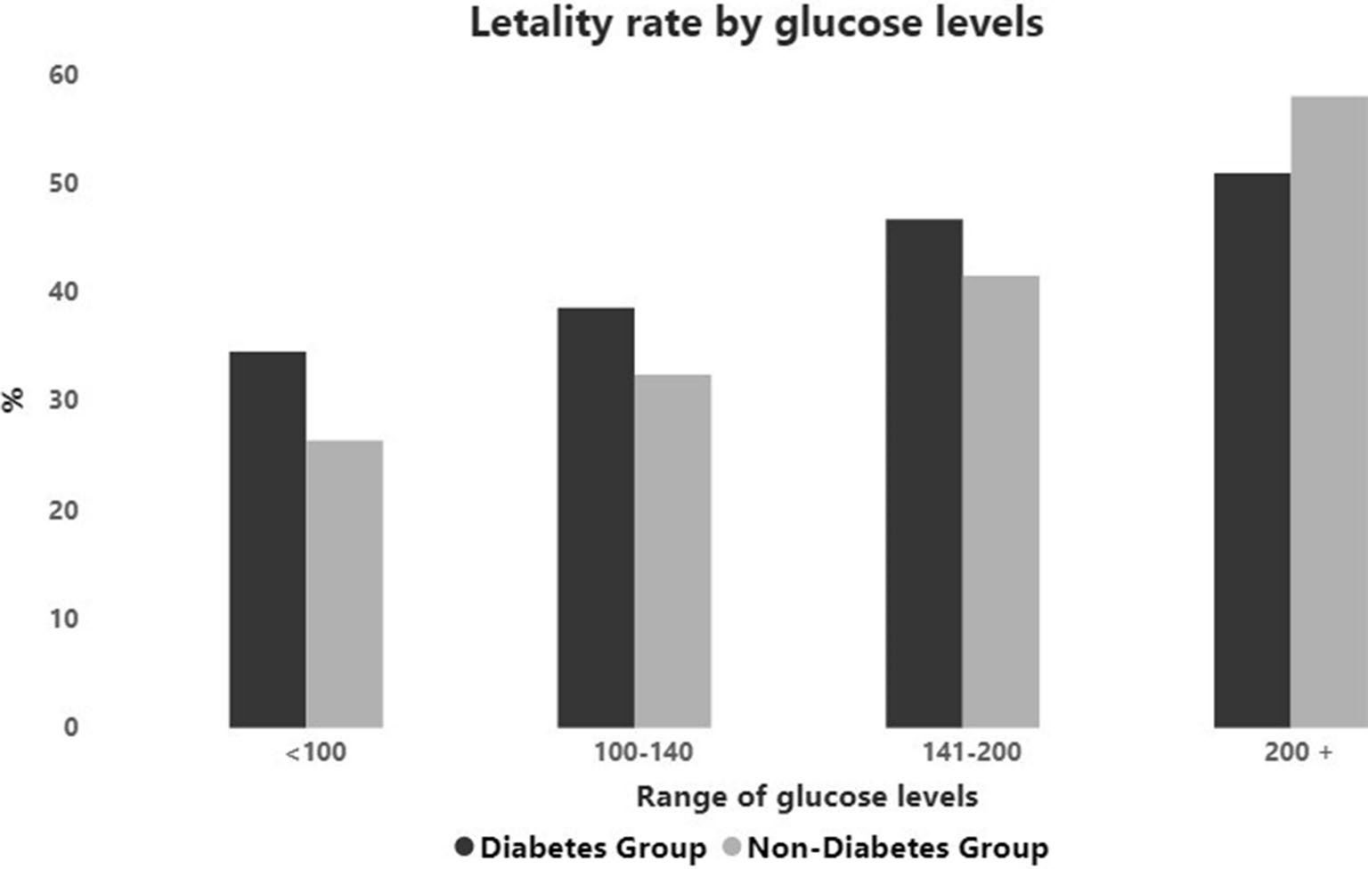
Lethality rate (%) by glucose levels at admission of patients with and without diabetes

Glucose levels also influenced the outcomes, with evident impact on the severity of COVID-19 in both groups. Levels greater than 126 mg/dL on admission were observed in 81.6% (626/767) of patents with diabetes but also in 50.3% (593/1179) of those without diabetes, being associated with a mortality rate of 49.04% and of 45.36% in DM and non-DM groups. A trend towards an increase in COVID-19 related lethality with the increase in glucose levels was noticed in both groups (Fig. 4) (Additional file 1: Table S14).

### Principal component analysis of four groups

The first group contained the standard measures obtained at patient admission (temperature, heart and respiratory rate, systolic and diastolic blood pressure and O_2_ saturation) presenting strong positive correlations among most of them (Additional file 1: Table S15), with the exception of O_2_ saturation (non-significant or negative with the other variables).

The principal component analysis of the correlations among these variables produced two uncorrelated variables (Adm PC 1 and Adm PC 2) that contain more than 50% of the overall variation and presented mainly positive correlations among variables (Additional file 1: Table S16).

Both principal components for parameters verified at patient admission were significantly different between patients that died, and patients released, suggesting mainly that lower systolic and diastolic blood pressure level (Adm PC1) and elevated temperature, heart and respiratory rate (Adm PC2) predisposed to death (Table 6). However, these variables were poor predictors of patient outcome, after a logistic regression analysis between principal components and outcome (Additional file 1: Fig S1A, B). The areas under the Receiver Operator Characteristic (ROC) curve for Adm PC 1 and Adm PC 2 were only 56.2% and 57.5% respectively and do not differentiated patients with and without diabetes (Table 7).

**Table 6:** Comparison between principal components of variables obtained at patient admission between the patient outcomes

	Condition	N	Mean	Standard deviation	Chi square	p value
Adm PC 1	Released	2176	0.09722	126.228	340.212	< 0.0001**
	Death	1117	− 0.18939	141.487		
Adm PC 2	Released	2176	− 0.09358	110.321	494.843	< 0.0001**
	Death	1117	0.18230	119.699		
INFLA PC 1	Released	843	− 0.24489	0.81823	2.065.813	< .0001**
	Death	452	0.45674	229.336		
INFLA PC 2	Released	843	− 0.19523	0.71300	1.553.776	< .0001**
	Death	452	0.36411	150.424		
COAG PC 1	Released	332	− 0.15639	0.88525	18.771	< 0.0001
	Death	287	0.18091	150.320		
COAG PC 2	Released	332	0.11805	0.84514	67.557	0.0093
	Death	287	− 0.13656	123.431		
REN PC 1	Released	1374	− 0.31957	133.508	1.903.586	< 0.0001
	Death	955	0.45978	165.429		
REN PC 2	Released	1374	− 0.06950	0.97101	82.911	0.0040
	Death	955	0.09999	124.439		

Critical p value = 0.025

Adm PC1 and Adm PC2, INFLA PC 1 and INFLA PC 2, COAG PC 1 and COAG PC 2 and RENAL PC 1 and RENAL PC 2-comprised principal Components (PC) of laboratory analyses related to admission, inflammatory, coagulation and renal status of patients

**Table 7:** Comparison between principal components of variables obtained a patient admission between patients with and without diabetes

	Condition	N	Mean	Standard deviation	t ratio	p value
Adm PC 1	Non-diabetes group	2080	− 0.02196	130.839	124.011	0.2151
	Diabetes group	1211	0.03780	134.742		
Adm PC 2	Non-diabetes group	2080	0.03132	113.504	− 20.622	0.0393
	Diabetes group	1211	− 0.05429	115.639		
INFLA PC 1	Non-diabetes group	780	− 0.01314	173.366	0.405236	0.6854
	Diabetes group	515	0.01990	119.900		
INFLA PC 2	Non-diabetes group	780	− 0.07849	119.645	3.386363	0.0007**
	Diabetes group	515	0.11888	0.89679		
COAG PC 1	Non-diabetes group	389	− 0.04242	0.99157	1.010123	0.3131
	Diabetes group	230	0.07175	153.530		
COAG PC 2	Non-diabetes group	389	− 0.07190	108.580	2.281311	0.0229
	Diabetes group	230	0.12160	0.97856		
REN PC 1	Non-diabetes group	1418	− 0.07666	152.799	3.078891	0.0021
	Diabetes group	910	0.12156	150.777		
REN PC 2	Non-diabetes group	1418	0.07749	104.787	267.289	< 0.0001
	Diabetes group	910	− 0.12128	115.375		

Adm PC1 and Adm PC2, INFLA PC 1 and INFLA PC 2, COAG PC 1 and COAG PC 2 and RENAL PC 1 and RENAL PC 2-comprised Principal Components (PC) of laboratory analyses related to admission, inflammatory, coagulation and renal status of patients

Critical p value = 0.025

The second group comprised laboratory analyses related to inflammatory status and tissue lesion: AST, ALT, Troponin, LDH, PKC, RCP levels and leukocyte number, with strong positive correlations among most of them (Additional file 1: Table S17). The resulting uncorrelated principal components were named INFLA PC 1 and INFLA PC 2 that contains more than 50% of the overall variation. INFLA PC 1 reflects positively all the variables and so INFLA PC2, except for ALT and AST levels (negative) (Additional file 1: Table S16).

The variables of INFLA PC1 and INFLA PC2 were predictors of death, especially elevated ALT, AST and LDH levels in INFLA PC1 and elevated RCP levels and leucocytes count in INFLA PC2, mirroring inflammation and tissue lesion (Table 6) (Additional file 1: Fig S1C–D). However only INFLA PC2 differentiated both groups, pointing to higher inflammation (elevated RCP levels and leucocytes count) and tissue lesion processes (high LDH and troponin levels) in patients with diabetes favoring death (Table 7).

The third group comprised laboratory analyses of coagulation related variables: PT, PTT, D-dimer and fibrinogen values) (Additional file 1: Table S18). The resulting uncorrelated principal components COAG PC1 and COAG PC2, containing more than 50% of the overall variation, reflected positively all the variables, except for fibrinogen in COAG PC1 and D-dimer in COAG PC2 (negative) (Additional file 1: Table S16). Both variables COAG PC1 and COAG PC2, related to activation of coagulation were predictors of death (Table 6) (Additional file 1: Fig S1E, F), but do not differentiated DM from non-DM group (Table 7).

The forth group comprised laboratory data of renal status and blood solutes obtained on admission: sodium, potassium, calcium, magnesium, urea and creatinine levels. The resulting principal components, RENAL PC 1 and RENAL PC 2 (Additional file 1: Table S19) contained more than 50% of the overall variation. RENAL PC 1 reflected positively all the variables except calcium, and RENAL PC2 all the variables except potassium and creatinine (negative) (Additional file 1: Table S16).

Both components of RENAL PC1 and RENAL PC2, expressing the worsening of renal status were predictors of death (Table 6) (Additional file 1: Fig S1G, H) and differentiated DM from non-DM group (Table 7), represented by elevated urea, creatinine, potassium in RENAL PC1 and reduced sodium and magnesium levels in RENAL PC2.

In the total analysis, variables of INFLA PC1, INFLA PC2 and RENAL PC1 had the greatest predictive power for death, as well as the index SAPS3 (Table 8) (Additional file 1: Fig S1I).

**Table 8:** Summary of predictive power of compound variables on the outcome (death or release) for patients with and without diabetes and for the total sample

Compound	Area under the ROC curve		Total %	O.R		Total
variable	DM Group %	Non-DM group %		DM Group	Non-DM Group	
SAPS3	72.60	75.20	74.50	0.0065	0.0002	0.0003
Adm PC 1	58.40	55.10	56.20	10.054	5.357	8.033
Adm PC 2	59.40	56.70	57.50	0.0924	0.155	0.108
INFLA PC 1	74.80	73.50	74.20	1.9 × 10^–5^	4.8 × 10^–12^	1.9 × 10^–12^
INFLA PC 2	69.90	70.70	71.00	8.9 × 10^–4^	1.6 × 10^–10^	1.1 × 10^–11^
COAG PC 1	58.30	61.50	60.10	0.0513	0.021	0.015
COAG PC 2	53.10	58.50	56.10	1.884	20.37	10.54
REN PC 1	71.10	63.40	66.80	2.7 × 10^–4^	0.027	0.026
REN PC 2	53.40	54.70	53.50	0.0565	0.115	0.186

Adm PC1 and Adm PC2, INFLA PC 1 and INFLA PC 2, COAG PC 1 and COAG PC 2 and RENAL PC 1 and RENAL PC 2-comprised Principal Components (PC) of laboratory analyses related to admission, inflammatory, coagulation and renal status of patients

DM, diabetes

## Discussion

This analysis focused on determining the main differential COVID-19 phenotypic manifestations of patients with diabetes in relation to those without diabetes. It comprises an extensive analysis of demographic data, pre-admission symptoms, clinical and laboratory characteristics on admission, treatment during the entire hospitalization and prognosis of COVID-19 in 3327 patients attended at a public hospital.

Diabetes was the second most common comorbidity after hypertension (57.4%), with 36.7% of patients suffering from the disease (Additional file 1: Table S1). Pre-existing comorbidities prior to admission, mainly hypertension, neurological and renal disease, older age and male sex predisposed to a more severe course of the disease and to death (data not shown) that occurred in 33.9% of the patients, and was higher in those with diabetes (39.2% × 30.7%; OR = 1.46; p < 0.0001) (Table 4). These data are in accordance with other epidemiological reports around the world [1–8] and in data from Brazil [9–13]

Men accounted for the majority of COVD-19 deaths in the total cohort—61.14% (689/1127), with greater risk both in men with (OR = 1.37) and without diabetes (OR = 1.50) (Table 4). This sexual dimorphism had been attributed to gender difference in immune response, higher expression of transmembrane serine protease 2 (TMPRSS2) promoted by androgen, facilitating SARS-COV-2 entry [19], frequent prevalence of comorbidities (hypertension, CVD, chronic lung diseases), occupational exposure, risk behaviors (smoking and alcohol), worse health habits and irregular medical appointments [20, 21].

However, the usual protection conferred by female gender seemed inappropriate in diabetes, as women with diabetes had lethality rates that do not differ to those of men without diabetes.: 35.3% × 34.5% (p = 0.7477).

Interesting, the frequency of death in patients with diabetes was similar to those without diabetes for each age range evaluated, (Fig. 2) and increased in a similar way with age, being 5 times more frequent in patients older than 80 years in relation to those lower than 40 years old, reaching 60% and over in the elderly. The fact that the presence of diabetes was not a determinant of mortality by age group may have depended to the large percentage of the population without diabetes that evolved with acute hyperglycemia, which was associated with a worse outcome. (Fig. 4). Deficiency of the immune system and other weaknesses associated with comorbidities, metabolic diseases and age sarcopenia probably contributed to the worse prognosis of patients with diabetes which were older than those of non-DM group.

Obesity deserves a special attention considering reported prominent effects on outcomes, provided by mechanisms like ventilatory difficult, insulin resistance, the hypercoagulable and pro-inflammatory environment, nutritional deficits and immune dysregulation, besides comorbidities, oxidative stress and lipotoxicity [22]

We also observed a trend to higher mortality in patients with diabetes versus those without diabetes for several BMI ranges that was significant only for those overweight (Fig. 3). However, in our cohort, obesity did not conditioned the worst prognosis, which prevailed in those with BMI lower than 30 kg/m2, appearing as a predictor of death in both groups (Additional file 1: Table S3, S6). Preexisting disease like neurologic and neoplastic diseases with illness-induced wasting and sarcopenia, smoking, drugs and inadequate glycemic control are possible predictors for the paradox of low BMI. Cariou et al. [17] also described the obesity paradox where the primary outcome was less pronounced in patients with morbid obesity compared with those who were overweight or with grade 1–2 obesity. The low frequency of obesity in our population (DM = 33.99%; non-DM group = 27.03%) may have influenced this result.

Clinical manifestations of COVID-19 prior to hospital admission did not differ between groups considering the median duration of COVID-19 symptoms (9 days) and the frequency of complains like dyspnea and cough (> 70%), followed by tiredness, muscle ache, gastrointestinal complains, headache and, to a lesser extent runny nose, odynophagia, anosmia, ageusia, mental confusion (Table 1). There were lower reports of fever in patients with diabetes, maybe due to dysfunction of the immune and of sympathetic systems, hyperglycemia and dehydration. This characterization is important particularly because it draws attention to the fact that they were hospitalized with a more severe condition despite similar previous symptoms that did not denote that severity.

Ethnicity and education level also did not impact on different outcomes in patients with and without diabetes (Table 5). Non-white ethnicity and low-education attainment usually contributes with several factors that worsen the prognosis like as low-income, lower access to quality healthcare, higher prevalence of comorbidities and poor nutrition, as observed in other studies in Brazil [10, 12]

However, our population assisted in a single public hospital was homogeneous in terms of cultural, ethnic (highly miscigenated population) and socioeconomic aspects which may have reduced the impact of these factor in different mortality rates between groups.

As expected, blood glucose levels on admission were higher among patients with diabetes (Table 2). Notwithstanding, hyperglycemia could not be considered exclusive of diabetes. It is important to point that glucose levels greater than 126 mg/dL were observed in 81.6% of DM-group but also in 50.3% of non-DM group, being associated with a lethality rate of 49.04% and of 45.36% respectively. There was a trend towards an increase in COVID-19 related mortality with the increase in glucose levels in both groups (Fig. 4 (Additional file 1: Table S14), evidencing the contribution of the severe infection to hyperglycemia. As suggested by Mondal et al. [16], the stress hyperglycemia, irrespective of the pre-existing glycemic status seems to worse the prognosis of patients and contribute to progression to critical disease, even in those without diabetes,

SARs-Cov-2 replication and cytokine production favored by elevated glucose levels [23], create an inflammatory condition that may affect pancreatic islet function and survival and cause insulin resistance, that coupled with the frequent administration of glucocorticoids probably accentuated the metabolic abnormalities.

Several markers of inflammation, tissue injury and organ dysfunction, mainly of cardiac and renal tissues were altered in both groups due to the severity of infection (Additional file 1: Table S2–S4,S6–S7), but more intensely in DM group (Table 2), presenting higher levels of CRP, LDH, troponin, PKC, lactate and BNP. Likewise, kidney damage was more prevalent among patients with diabetes, evidenced by higher blood levels of urea, creatinine, sodium, potassium and lower of albumin (Table 2). They also presented lowers levels of magnesium which have previously been implicated in lower survival of COVID-19 patients, given its relevant role in the homeostasis of the immune system, in the metabolism of carbohydrate and transport of calcium and potassium [23].

Interesting, the higher lethality of group with diabetes was not accompanied by expressive difference in their clinical manifestations, symptoms and laboratory values in relation to non-DM group, except for hyperglycemia and data of renal dysfunction,. Not even difference in leukocyte count (or neutrophils and lymphocytes- data not shown) and clotting markers were evidenced. (Additional file 1: Table S9–S11), suggesting that the higher mortality seemed to depend on several factors. In order to precise the main factors involved in outcomes, we analyzed the impact of a set of characteristics on disease progression. The principal components (PC1 and PC2) related to clinical characteristics on admission and to factors related to inflammation, renal function and coagulation were all predictors of death in the total population (Table 6), except for PC2 of coagulation and of renal factors (Table 6). However, only INFLA PC2 containing inflammatory factors (expressed by higher leucocyte number, RCP, LDH and troponin and lower AST and ALT levels) and mainly, components of renal function- (higher urea, creatinine and potassium levels in RENAL PC1 and lower sodium and magnesium in RENAL PC2) differentiated lethality risk between patens with and without diabetes (Table 7). Considering the area under the ROC curve (Additional file 1: Fig S1), both worsening in renal function and elevated levels of inflammatory factors were highly predictive of death (areas greater than 65%), but especially Inflammatory factors, which agree with reports of the harmful effects of the cytokine storm, probably potentiated by the pro-inflammatory state of patients with diabetes (ref). These components were even more specific predictors of death than SAPS3 index (Table 8). As the principal components are based on linear correlations, any non-linear correlations, which are frequent in pathophysiological phenomena, may not be verified. Thus, some physiological interpretations of principal components may not have been evident for this reason.

In addition, the poor prognosis of COVID-19 in patients with diabetes was also associated with previous comorbidities. They have almost twice the frequency of hypertension, CVD, CKD and previous stroke in relation to those without diabetes (Additional file 1: Table S9) which probably contributed to the risk of severe COVID-19, as reported by Landstra et al. [8], considering comorbidities and risk factors normally present in patients with diabetes.

Another factor to account in our results was the previous treatment with RAAS blockers, which was associated with lower mortality in both groups, indicating that ACEi/ARBs are not risk factors for disease severity (Table 5). On the contrary, previous use of ACEi/BRA seemed to protect patients with diabetes, which lethality was similar to those without DM (33.53 × 30.08; OR 1.172; CI 0.8986–1.530; p = 0.2501). Otherwise, lethality among patients with diabetes without previous RAS inhibitor treatment was higher in comparison with those without DM (48,95% × 37.51%; OR 1.597; CI 1.268–2.011; p < 0.0001). A neutral effect of these medications in COVID19 patients with diabetes [17] or a protective [24] or aggravating [25–27] effect in general population has been reported.

In addition, it is important also to consider clinical deficiencies or treatment previous to hospitalization in our center that may have precluded a better differentiation of both groups like administration of catecholamines, glucocorticoids, anti-coagulants, antibiotics, other drugs, dialysis and inadequate fluid therapy.

Some limitations have to be considered. All the data were from a single public center and might not reflect the conditions found at private counterparts. Diabetes subtypes were not described due to limited data of the diagnosis. Moreover, laboratory data were not available for all patients. Previous treatment of patients before admission may have interfered with the results.

Strengths of our analysis included detailed description of COVID manifestations in a sizable cohort of patients with diabetes, including symptoms, clinical and laboratory manifestations, long-term outcomes and prescriptions among hospitalized patients in relation to those without diabetes. All the laboratory analyzes were done at the same center and patients were treated with the institutional protocols of the largest health care center in Brazil. All these parameters have been little explored in diabetes in previous reports.

In conclusion comorbidities, inflammatory markers and renal disfunction, age, male sex but not Covid-19-related symptoms, obesity, ethnicity and education level differentiated lethality risk between patients with and without diabetes.

### Supplementary Information

**Additional file 1: Table S1.** Data at admission, laboratory data and treatment. **Table S2.** Frquency of hospital permanence. **Table S3.** Clinical data of COVID-19 patients with diabetes on hospital admission. **Table S4.** Laboratory data of COVID-19 patients with diabetes on-admission. **Table S5.** Treatment data during hospitalization of COVID-19 patients with diabetes. **Table S6.** Clinical data of COVID-19 patients withou diabetes on hospital admission. **Table S7.** Laboratory data of COVID-19 patients without diabetes on-admission. **Table S8.** Treatment data during hospitalization of COVID-19 patient withou diabetes. **Table S9.** Clinical data of COVID-19 patients on hospital admission that went to death. **Table S10.** Laboratory data of COVID-19 patients that went to death on-admission. **Table S11.** Treatment data during hospitalization of COVID-19 patients that went to death. **Table S12.** Frequency of according to age. **Table S13.** Frequency of death according to body mass index. **Table S14.** Frquency of death according to glucose levels. **Table S15.** Multivariate correlations among standard variables obtained at patient admission. **Table S16.** First two principal components from the inflammation related variables obtained at patient. **Table S17.** Multivariate correlations among inflammation related variables obtained at patient admission. **Table S18.** Multivariate correlations among coagulation related variables obtained at patient admission. **Table S19.** Multivariate correlations among variables related to renal function obtained at patient. **Figure S1.** Receiver Operator Characteristic (ROC) curves on the outcome death/release of the laboratory data obtained at patient at admission Adm PC 1 (**A**) and Adm PC2 (**B**).

## Data Availability

All data generated or analyzed during this study are included in this published article (and its Additional file 1: Table S1–S19) and are available upon request to the corresponding author.

## Abbreviations

ACEI/BRA: ACE inhibitors and/or angiotensin II receptor blockers
ADM PC: Principal Component at admission
ALT: Alanine aminotransferase
AST: Aspartate aminotransferase
BNP: Brain natriuretic peptide.
CKD: Chronic kidney disease and/or dialysis
COAG PC: Principal component related to coagulation
COVID-19: Corona virus disease 2019
CPK: Creatine phosphokinase
CRP: C-reactive protein (CRP)
CVD: Cardiovascular Disease
INFLA PC: Principal component related to inflamation
LDH: Lactate dehydrogenase,
PT: Prothrombin time
PTP: Partial thromboplastin time
RENAL PC: Principal component related to renal status
SAPS 3: Score simplified acute physiology III
